# Report of a Case of Triplets

**Published:** 1846-11

**Authors:** John F. Hammond

**Affiliations:** Silverton S. C.


*Report of a case of Triplets.* By John F. Hammond, M. D., of Silverton S. C.—Mrs. S——, was taken with symptoms of labor, August 26th, at 5 o'clock, A. M. I was called to her at 4, P. M., and found, lying on a pallet, a woman above the average size, bilious temperament, and who had had nine well formed, healthy children. Having been in labor for thirteen hours, and in the care of a colored midwife, she was necessarily discouraged, and the uterus had begun to flag, though not from want of capability. I found all else in an admirable state; pulse good, head clear, bowels just evacuated, bladder empty; the vertex in the first position, (Velpeau,) and the os tincæ fully dilated; the bag of waters projected largely into the vagina. A few words revived her spirits and restored her energy; she was placed upon her side. I ruptured the membranes with my thumb and finger nails, which, from their toughness, I found very difficult to do, and a few minutes after six o'clock, the second stage (Denman) was completed; she gave birth to a fine boy. He was removed, and a second was found presenting by the breech, in the fourth position; the bag of waters soon formed and was ruptured, and at half-past seven she was delivered of a girl; the head passing in the second position, the change occurring spontaneously, there was no necessity for interference. Up to this time there was not a drop of hemorrhage, except from between the ligatures around the cords when the cords were severed. On examination, a third fœtus was discovered in the first position of the breech. There was now an intermission of the pains of some minutes' duration, and their recurrence during the remainder of the labor was less frequent, though not less protracted; and though wearied, she was cheerful. Giving time for the bag of waters to form, it was ruptured, the breech passed, and the shoulders with the trunk carried over the mother's abdomen, two fingers against the occiput, and two eliciting it upon the upper maxillaria, the head was delivered in the first position before eight o'clock, after full half a minute's compression of the cord in passing the inferior strait. The cord was unusually short. A little hemorrhage followed. The uterus being free of all but the placentæ, the saturated vinous tincture of ergot was given freely, kneeding, cold wet cloths, compresses and a bandage were employed on the abdomen and gentle traction by the cords; blood was flowing, though not copiously; the foot of the pallet was elevated by pillows, her head lowered; the placentæ perfectly attached to each other, passed into the vagina and from the vulva in about forty minutes after the delivery of the last fœtus. The ending of the third stage was followed by a profuse flooding, which in a few minutes prostrated the patient almost completely. By the former means and titillation of the uterus, it contracted rapidly; she rallied, the pulse revived, and in a few minutes she was carefully placed in bed, with her hips elevated. The sixth of a grain of sulph. morphia soon induced a quiet sleep, andanother dose relieved a few pains which came on in the course of the night. The next morning, she was doing well; had slept well—but little discharge. The children are all well. The following are their several weights:—1st male, 5 lbs.; 2d, female, 4 lbs.; 3d, male, $4\frac{3}{4}$ lbs. Total, $13\frac{3}{4}$lbs.

These weights appear small, but upon re-weighing, they were found correct. The 1st of September, being the 7th day, mother and children were all doing well.

Note.—The extreme rarity of triplets, and the great particularity with which Dr. Hammond describes the process of parturition in this case, renders the above communication very interesting. Triplets do not occur oftener than about one in six thousand cases. The late Professor Dewees, in his extensive practice in the city of Philadelphia, met with only one case. We only know of two cases ever having occurred in this city—one in the practice of the late Professor Antony, some twenty or twenty-five years ago—the other, in the practice of the present Professor of Midwifery, in 1837 : in the latter case, the heads of the second and third child became locked in their passage through the pelvis, and were expelled together. An account was given in the 2d volume, 1st series of this Journal, p. 180.—Eds.

*Med. and Sur. Journal.*

